# Supplementary material for: Mining yeast diversity unveils novel targets for improved heterologous laccase production in Saccharomyces cerevisiae
Source: Microb Cell Fact. 2025 Mar 10;24:60. doi: 10.1186/s12934-025-02677-1 (PMC11892151; doi:10.1186/s12934-025-02677-1)

**Fig. S1. Development of the Laccase Secretion Assay and comparison of Laccase Structure and Sequence.** **A)** Plasmid map of BPM1747 with laccase. **B)** Laccase activity from BY4741 cells expressing and secreting ttLcc1 was measured over a 7-day period. **C)** Heatmap plotting relative laccase activity from BY4741 cells expressing ttLcc1 for 4-days in a 96-deep-well plate. **D and E)** Ecological origin distributions of the full library (D) and of the 597 transformed strains from which the laccase activity was assessed (E). **F)** Structural alignment of ttLcc1 (blue, PBD: 2HRG) and mtLcc1 (brown, PBD: 6F5K). **G)** Protein sequence alignment of ttLcc1 and mtLcc1 highlighting sequence identity.


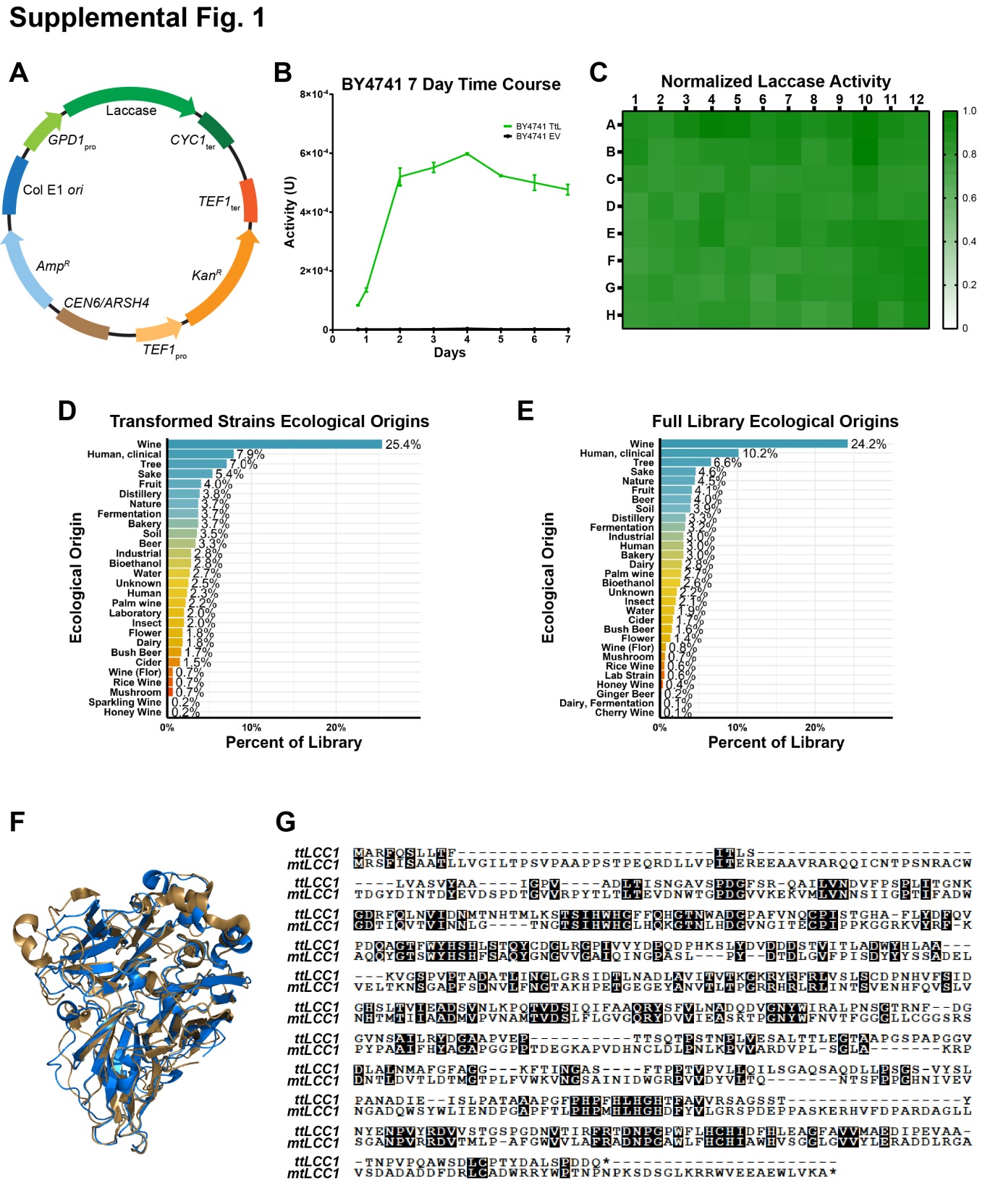


**Fig. S2. Comparison of Relative Strain Growth and *ttLCC1* mRNA Levels with Laccase Activity.** **A)** Relative cell density (represented by bars) and laccase activity (points) in the indicated strains expressing *ttLCC1* after 4-days growth in comparison to BY4741 for the experiment shown in Fig. 1C. **B)** Laccase activity for 3 of the hit strains grown in a 25mL culture was assessed over 7-days. Activity from BY4741 cells shown in Fig. S1B was replotted for comparison. **C)** *C_T_* profile of the internal reference *UBC6* and target *ttLCC1* mRNA in the indicated strains. **D)** Melting curve of *ttLCC1* target demonstrates a single target was amplified.


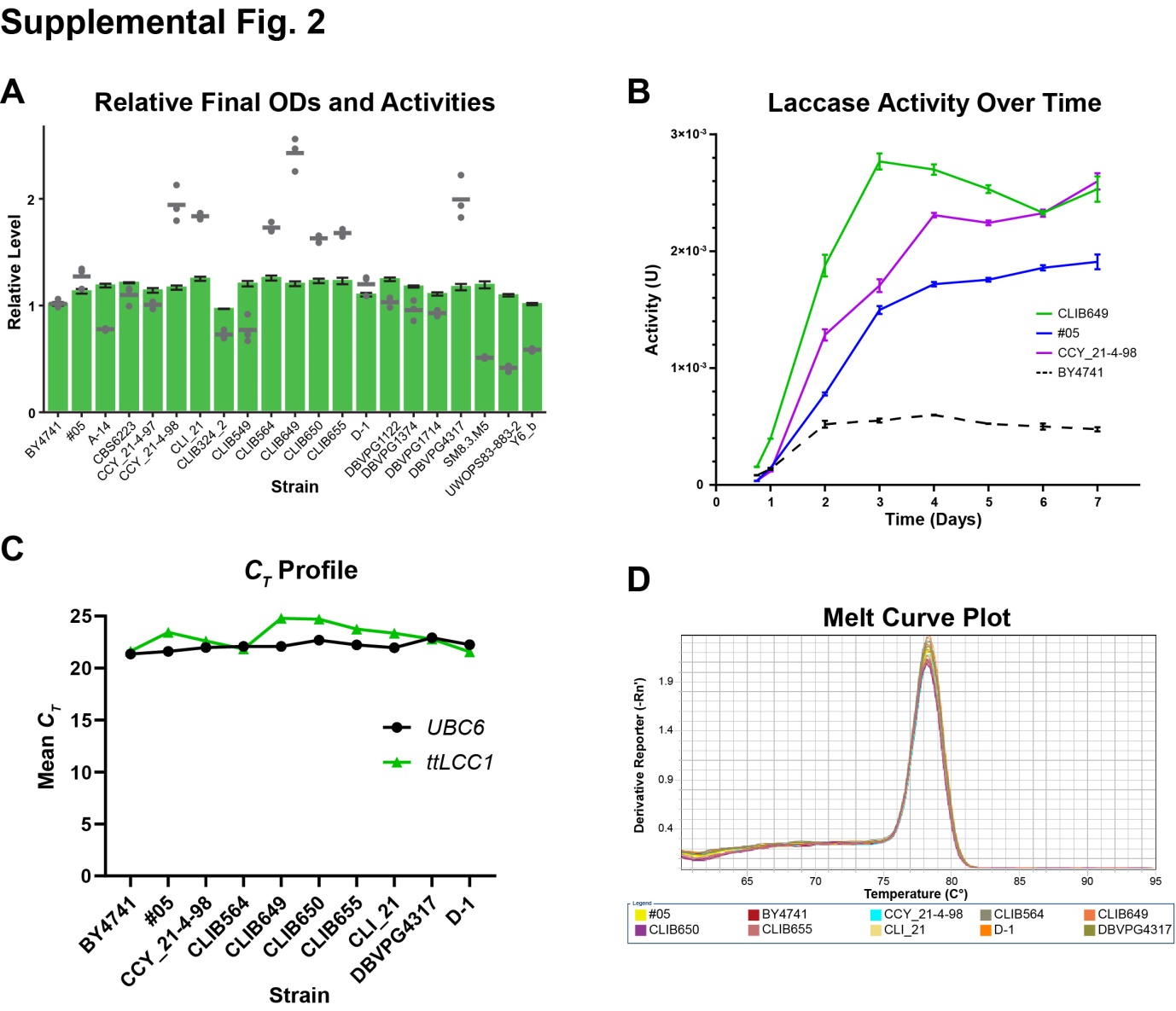


**Fig. S3.** **Glycosylation may reveal why ttLcc1 Activity is Higher in CLIB649. A)** ttLcc1 stability at room temperature assessed over 12 days. **B)** Activities and yields of ttLcc1 purified from BY471 and CLIB649 (p-values: * < 0.05, ** < 0.01 *** < 0.001, **** < 0.0001). **C)** ttLcc1 with and without PNGase F treatment to remove glycosylation, visualized by stain-free polyacrylamide gel. Glycosylation-free ttLcc1 is expected between 55–56 kDa, while glycosylated ttLcc1 is estimated at ~ 65 kDa.


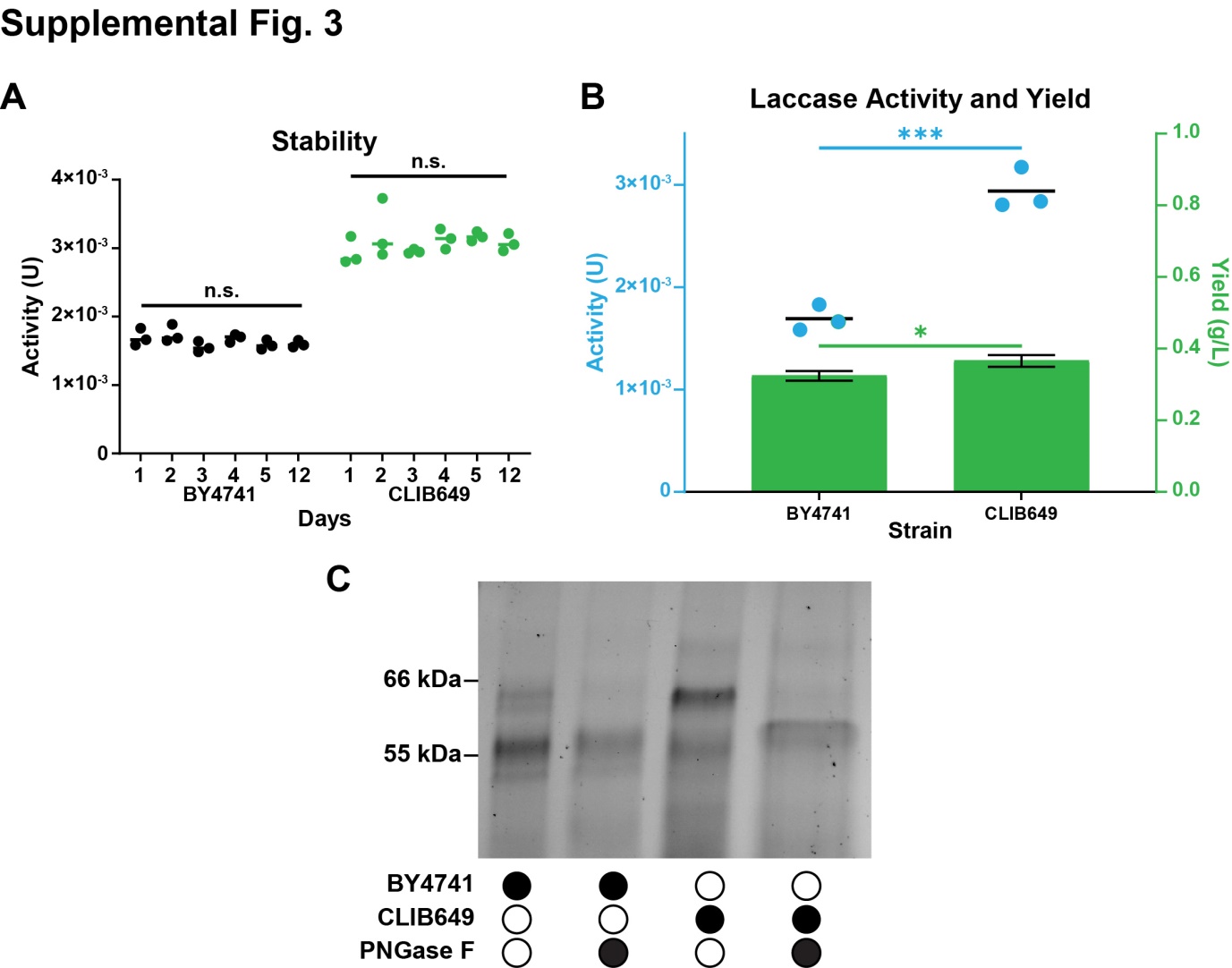


**Fig. S4. SNP and CNV GWAS analysis in Laccase Activity in 597 Strains.** **A)** SNP GWAS identified a single significant SNP in *ATG27/ETF1* of chromosome 15. **B)** CNV GWAS identified a single significant CNV in the locus encompassing YPL273W and YPL274W on chromosome 16.


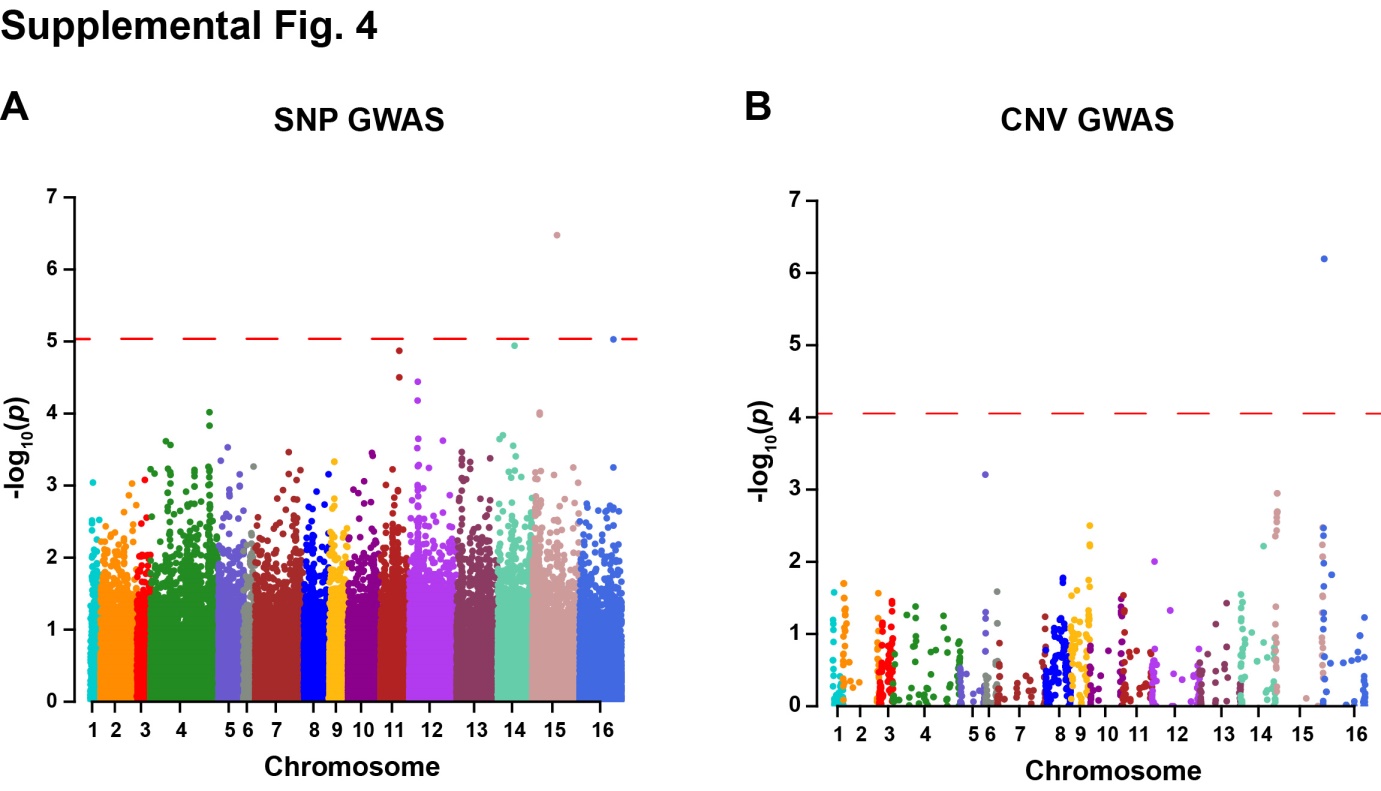


**Fig S5. *hoΔ* Laccase Activities.** Laccase activity of individual clones after 4 days growth (n = 4; p-values: * < 0.05, ** < 0.01 *** < 0.001, **** < 0.0001).


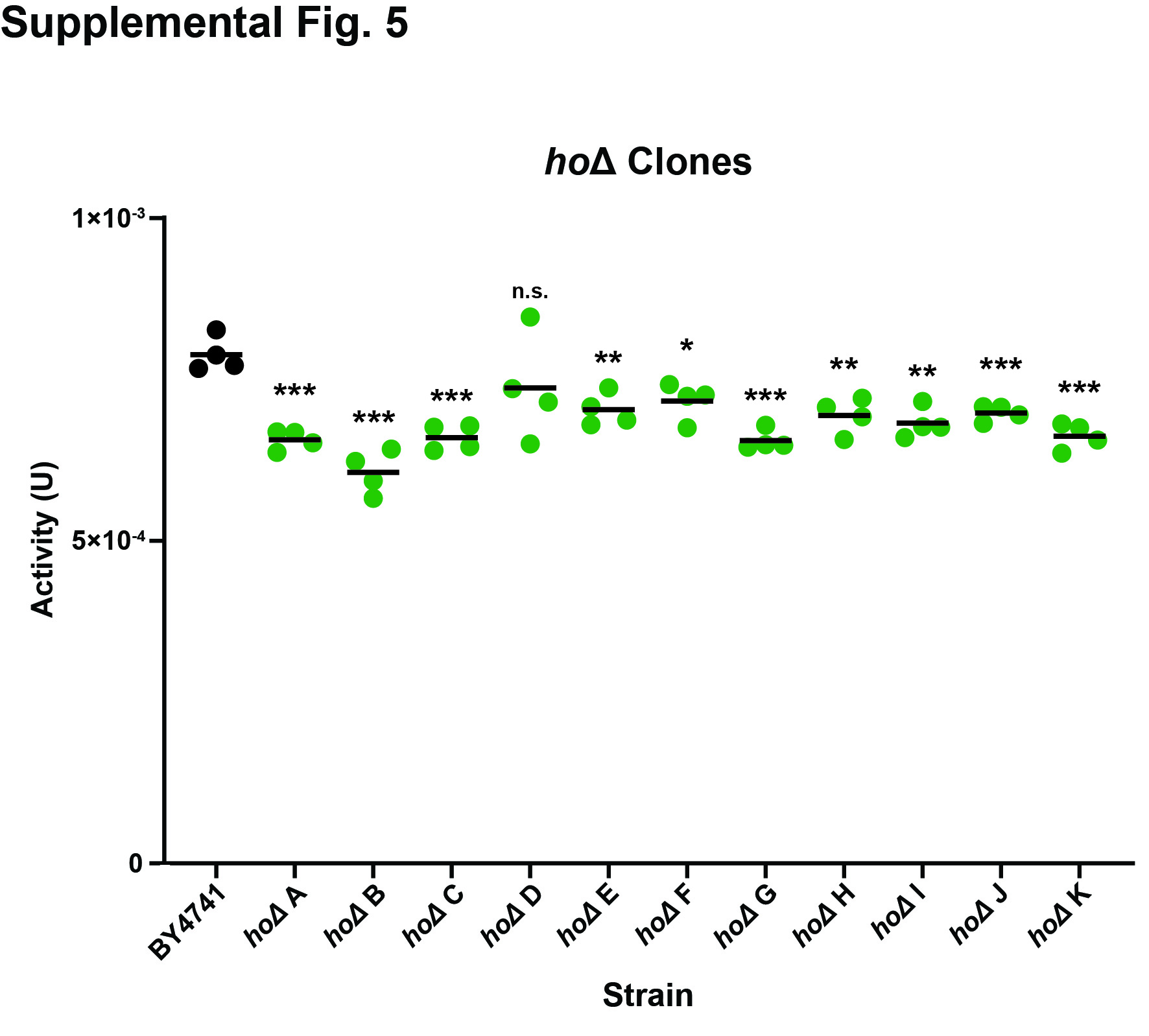


**Fig. S6. Proteomics Data Quality Control and Intracellular Laccase Levels of Select Strains.** **A)** Correlation heatmaps of strains A-14, D-1 and Y6_b. Replicates indicated in red were removed from further analysis due to low correlations indicative of potential errors during sample preparation. **B and C)** CV density plots of the technical replicates of one BY4741 sample (n = 14) and biological replicates (n = 7) of BY4741 cells grown in different wells. Median CVs shown (green dotted lines). **D)** Scatter plot of CVs of each protein identified in each mass spectrometry run of the 20 hit strains and BY4741 against its averaged intensity. CVs steeply increase for some proteins with low intensities. **E)** Plot of the median CVs of the 20 hit strains. **F)** Correlation plots of selected strains. Only proteins quantified in both strains are shown after averaging their intensities from the biological replicates. Pearson correlation indicated. **G)** The number of significantly enriched and depleted proteins in the indicated number of strains in comparison to BY4741. 1565 proteins are found to be enriched in at least 1 of the strains, down to a single protein enriched in all 20 strains. Similarly, 1481 proteins are depleted in at least 1 of the strains and 8 proteins are depleted in all 20. **H)** Relative intracellular ttLcc1 intensities of the indicated strains after 4-days growth (BY4741: n = 7; D-1: n = 3; remaining: n = 4; p-values: * < 0.05, ** < 0.01 *** < 0.001, **** < 0.0001).


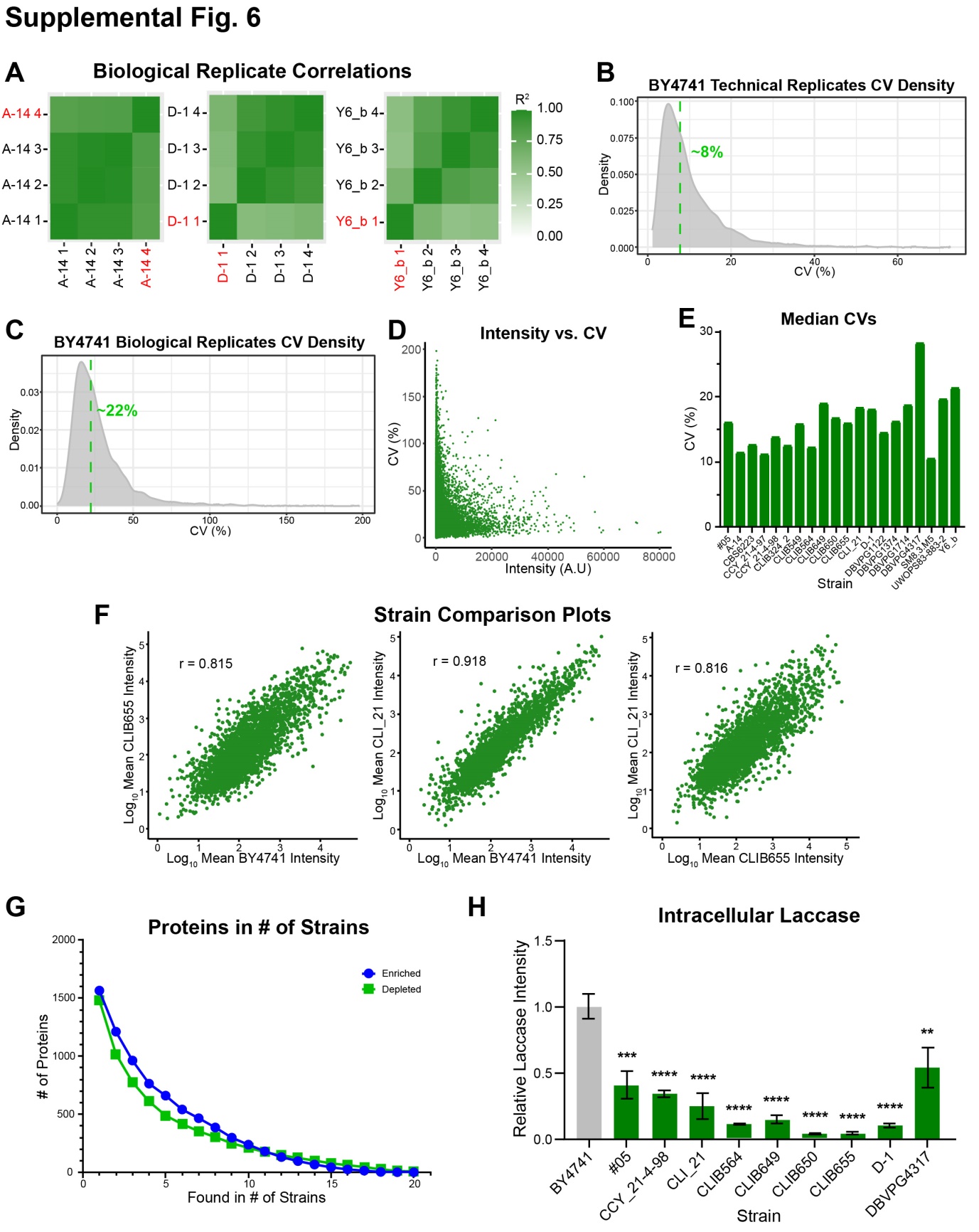


**Fig. S7. Comparison of Laccase Activity in *prm8Δ* and *prm9Δ* strains.** Laccase activity in the indicated strains expressing ttLcc1 after 4-days growth (n = 4; p-values: * < 0.05, ** < 0.01 *** < 0.001, **** < 0.0001).


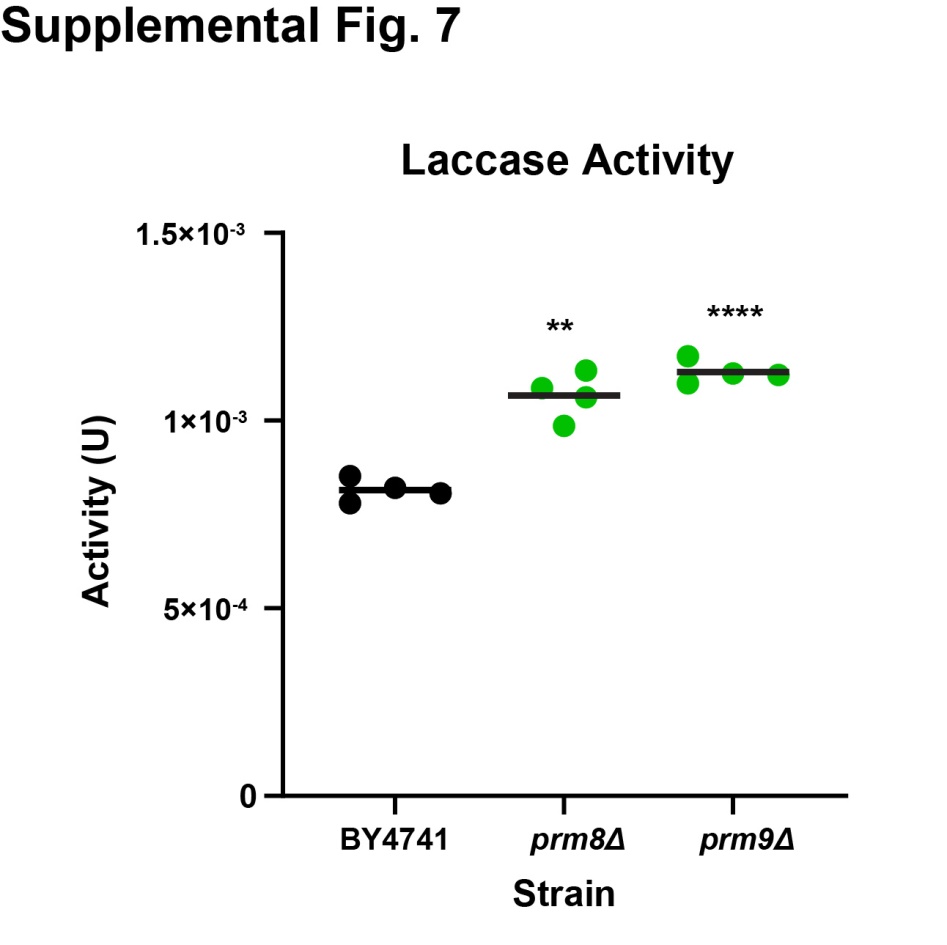

Supplement: Supplementary file 5 — Supplementary Material 5 [file 12934_2025_2677_MOESM5_ESM.docx]
